# 

**Published:** 1905-05-15

**Authors:** 


### ANNOUNCEMENTS.

THE Indiana State Dental Association will meet in Indianapolis at the Claypool Hotel, June 27 to 29, 1905. The failure to organize the expected tri-state convention this year has made it necessary to provide for a State meeting this year. The committee is hard at work and promises one of the best State meetings ever held. The meeting and exhibits will be held on the same floor. Let everybody come prepared to have a good time.

A. G. WHITE, *Sec.*
New Castle, Ind.

MICHIGAN STATE DENTAL ASSOCIATION.—This year's meeting will convene in Detroit on the 10th, 11th and 12th of July. It is the purpose of the directors to present this year a program exclusively of home talent, and it will be largely of the clinical order. The papers will be short and of the most practical character. A large number of clinics are already promised and more will be secured by the time of the meeting. The meetings will be held in the Dental Department of the Detroit College of Medicine, so that every clinical advantage will be available. The Detroit Dental Society has the matter of entertainment in hand, and one evening and one afternoon will be given over to social features. It is the intention to outdo the famous tri-state convention, if possible.

---

Ohio State Board of Dental Examiners.—The regular semi-annual meeting of the Ohio Board of Dental Examiners will be held in Columbus, June 27, 28, and 29, 1905. at the Hartman Hotel.

Applications for examination should be filed with the secretary by June 17th. For further information address,

H. C. Brown, *Sec.*
185 E. State St., Columbus, O.

---

National Dental Association.—Ninth annual session to be held in Buffalo, N. Y., July 25th to 28th, inclusive.

The Hotel Iroquois has been selected by the local committee of arrangements, as headquarters, where all general sessions of the association and of the sections will be held. The clinics will be held at the rooms of the dental department, University of Buffalo.

Rates at the Hotel Iroquois are single room per day, \$1.50, \$2.00 and 2.50; rooms for two persons, \$3.00 and \$4.00; single rooms with bath, \$3.00 and \$3.50; rooms with bath for two persons, \$5.00, \$6.00, \$7.00 and \$7.50; all rooms on the European plan.

The usual railroad rate of one and one-third fare for the round trip, certificate plan, has been arranged by the executive committee.

All pay full fare going taking the proper certificate therefor from the ticket agent, which, when properly viseed at the meeting, entitles the holder to return for one-third the regular rate.

Tickets going may be purchased from July 20th to 26th, and are good returning to and including August 2nd.

Both the general officers and those of the sections have been working hard to provide an interesting and instructive program and a large attendance is expected.

A. H. PECK, *Rec. Sec.*
92 State St., Chicago, Ills.

—✦—

THE Central Michigan Dental Association held a good meeting at Lansing, March 29th and 30th, but the attendance was not as large as it should have been. R. W. Morse, of Lansing, was elected president, C. W. Horning, of Portland, Vice-president, and A. C. Carr, of Lansing, Secretary and Treasurer.

—✦—

THE forty-sixth annual meeting of the Northern Ohio Dental Association will be held June 6th, 7th and 8th, at Gray's Armory, Cleveland, Ohio.

This is not only one the of oldest, but is one of the very best attended meetings in the country. This year the program is one of unusual strength and interest. The leading subjects for consideration are:

1. Humanitarian Methods.
2. Mistakes.
3. Prophylaxis.

Under the first is considered High Pressure Anesthesia, by Dr. C. G. Myers, of Cleveland; and High Pressure Anesthesia as Compared with other Pain Preventing Methods, by Dr. D. H. Zeigler, of Cleveland.

Essays under the second group include the Mistakes of the Country Dentists, by Dr. R. D. Wallace, Scio, Ohio; Mistakes of the City Dentists, by Dr. F. J. Spargur, Cleveland, Ohio; and Mistakes in Ethics, by Prof. S. H. Guilford, of Philadelphia, Pa.

The third includes the essays: Two Sources of Tooth Life and their Relative Importance, by Dr. D. D. Smith, of Philadelphia, Pa.; and Diseases of the Peridental Membrane and Treatment, by Dr. J. V. Stahl, of Wooster, Ohio.

The essayists and those who open discussion upon the various papers, have been selected for their particular fitness to handle subjects assigned to them.

Under Mistakes in Ethics, Dr. Guilford will point out, as only he can, some mistakes that are being made by the profession in the relation of its members to each other, together with the mistakes made in treatment of patients and the public. Great good is expected to result from the presentation of this paper and the discussions that follow. Many false impressions have existed in the past and still exist as to the duties we owe to each other, our patients and the public, and it is expected that the three papers on mistakes will do much to correct this.

Dr. Smith's paper bears upon that all-important subject, prophylaxis; he will bring a patient with him showing results accomplished by his method of procedure. He will illustrate his paper with models and instruments.

Throughout the entire program, much attention will be given to the study of Humanitarian Methods (methods which make it possible to perform dental operations free from pain).

The two papers, Application of High Pressure Anesthesia, and High Pressure Anesthesia as Compared with other Pain Preventing Methods, and the discussions to follow, will set forth all that is known of importance in this connection.

There will be about fifty clinics selected and arranged to give the knowledge seeking dentists the best post-graduate course that can possibly be obtained in a three day's meeting. One session will be devoted to the study of manufacturers' exhibits. The exhibits this year are to be one of the interesting features of the meeting, and the committee has been promised one of the largest exhibits shown in the country.

All communications pertaining to clinics or exhibits should be addressed to the corresponding secretary, Dr. W. G. Ebersole, 800 Schofield Building, Cleveland, Ohio.

Special rates of a fare and a third have been granted on the certificate plan by the Central Passenger Association.

The committe extend a most cordial invitation to the members of the profession to attend.

W. G. EBERSOLE, *Sec.*

---

NATIONAL ASSOCIATION OF DENTAL EXAMINERS.—The annual meeting of the National Association of Dental Examiners will be held at the Iroquois Hotel, Buffalo, N. Y., commencing at 10 o'clock Monday, July 24th, 1905, and continuing until adjourment.

The sessions will be held in commodious rooms in the hotel. Write early and secure your rooms. It is earnestly requested that secretaries of the boards will communicate as once any change in members' names and address.

CHARLES A. MEEKER, D.D.S., *Sec.*
29 Fulton St., Newark, N. J.

---

NEW JERSEY STATE DENTAL SOCIETY.—The thirty-fifth annual meeting of the New Jersey State Dental Society will be held in the Auditorium, Asbury, Park, N. J., commencing July 19th, and continuing until July 22d. Meeting commences promptly at 10 a. m. on the 19th. The various committees have been successful in securing eminent practitioners for papers of present interest, and some fifty clinicians in the most modern up-to-date dentistry. The space in the large Auditorium will be almost filled with all the newest appliances to practice dentistry.

Friday evening will be devoted to the social side with a smoker, including a collation and entertainment to the guests, exhibitors and members. Cut out now the week of July 17th and meet with us.

Seven hundred and fifty-six dentists registered last July—make it a thousand this year.

CHARLES A. MEEKER, *Sec.*

Newark, N. J.

—✦—

SOUTHERN INDIANA DENTAL ASSOCIATION.—TheSouthern Indiana Dental Association was organized at Paoli, March 10th, 1905, and the following officers were elected: President, E. S. Denbo, Orleans; Vice-president, C. D. Driscol, Paoli; Secretary, J. D. Vanosdol, Mitchell; Assistant Secretary, F. E. Woods, French Lick Springs; Treasurer, C. B. Tattershall, Paoli.

—✦—

ODONTOLOGICAL SOCIETY OF WESTERN PENNSYLVANIA.—The Odontological Society of Western Pennsylvania held its annual meeting at Pittsburg, March 14th, 1905, and elected the following officers: President, W .S. Cook, Beaver Falls; Vice-president, W. L. Haines, Pittsburg; Secretary, B. M. Loar, Mt. Pleasant; Treasurer, J. A. Libbey, Pittsburg; Executive Committee, C. C. Taggart, Pittsburg; M. S. Burns, Sewickley; D. W. Flint, Pittsburg.

—✦—

ANY OLD THING IN DENTISTRY.—One of the features of the next annual meeting of the New York State Dental

Society, to be held at Albany, May 12th and 13th, 1905, will be an exhibit of antique things in dentistry—instruments, pictures, appliances, abnormal models, in short, any old thing in dentstry. If you have any such, bring it with you, or if unable to come, send it with history to Dr. J. L. Appleton, chairman of arrangements, 89 Columbia street, Albany, who will see that it is returned to the owner in as good condtion as when received.

J. L. APPLETON.

---

AMERICAN MEDICAL ASSOCIATION—SECTION ON STOMATOLOGY.—The next meeting of the American Medical Association will be held in Portland, Oregon, July 11th to 14th, 1905. The program for the Section on Stomatology is as follows:

1. Chairman's Address, Vida A. Latham, Chicago.
2. The Causes and the Treatment of the Mouth Manifestations of Certain Metabolic Disorders, Alfred C. Croftan, Chicago.
3. The Oral Manifestations of Diabetes Mellitus, Herman Prinz, St. Louis.
4. The Urine and Saliva in So-called Pyorrhea Alveolaris, Wm. J. Lederer, New York.
5. Further researches in the Treatment of Interstitial Gingivitis, Eugene S. Talbot, Chicago.
6. Excretion of Toxic Products into the Mouth with Relation to Local Infection, Fenton B. Turck, Chicago.
7. The Relations of Dentistry to General Medicine, Samuel A. Hopkins, Boston.
8. A Common Ground for Medicine and Dentistry, Frank L. Platt, San Francisco.
9. The Physician as a Dentist, Calvin W. Knowles, San Francisco.
10. The Physician's Duty to the Child from a Dental Standpoint, Alice M. Steeves, Boston.

11. Dentistry of To-morrow, H. P. Carlton, San Francisco.

12. What will Probably be the Dental Educational Standard for the Coming Decade? C. C. Chittenden, Madison, Wis.

13. Fatal Oral Pathologic Conditions, G. V. I. Brown, Milwaukee.

14. Surgical Bacteriology of the Mouth, A. H. Levings, Milwaukee.

15. Surgical Aspects of Disturbed Dentition of the Third Molar, M. L. Rhein, New York.

16. The Treatment of Suppurative Affections of the Face and Neck Emanating from the Mouth, M. I. Schamberg, Philadelphia.

17. The Medical Relations of Certain Conditions of the Mouth, L. Duncan Bulkley, New York.

18. Some Effects of Inebriety on the Teeth and Jaws, T. D. Crothers, Hartford, Conn.

19. The Ossification of the Lower Jaw, Edward Fawcett, Bristol, Eng.

20. Ankylostomiasis and Tongue Pigment, T. M. Russell Leonard, Grenada, British West Indies.

21. Notes on Tooth Genesis in Man, H. W. Marett Tims, London, Eng.

22. The Etiology of Tooth Corrugations, G. Lenox Curtis, New York.

23. To What Extent are Teeth Necessary to the Human Being? M. H. Fletcher, Cincinnai.

24. Anesthesia by Ethyl-Chlorid and Similar Agents, H. C. Miller, Portland, Oregon.

25. The Rontgen Rays in Dentistry, M. Kassabian, Philadelphia.

The program is entirely scientific. All dentists are invited to be present and take part in the discussions. Those wishing to become members may do so by filling out blanks furnished by the Association, signed by the president of State or local dental or medical society, enclose five dollars and send to the Secretary on Stomatology for his signature. This also includes the Journal of the American Medical Association for one year.

EUGENE S. TALBOT, *Sec.*
Chicago, Ills.

---

INTERNATIONAL DENTAL FEDERATION.—The next annual meeting of the Executive Council of the Federation Dentaire Internationale will convene in Hanover, Germany, August 7th, 1905, immediately following the annual meeting of the Central-Verein Deutscher Zahnarzte. Announcement of the program for the meeting and the projected work for the Federation during the present period will shortly be made through the dental journals and through the official bulletin of the Federation.

EDWARD C. KIRK, *Sec.-Gen.*

